# The MetJ regulon in gammaproteobacteria determined by comparative genomics methods

**DOI:** 10.1186/1471-2164-12-558

**Published:** 2011-11-14

**Authors:** Anne M Augustus, Leonard D Spicer

**Affiliations:** 1 Department of Biochemistry, Duke University, Durham, North Carolina, 27710, USA; 2 Department of Radiology, Duke University, Durham, North Carolina, 27710, USA

## Abstract

**Background:**

Whole-genome sequencing of bacteria has proceeded at an exponential pace but annotation validation has lagged behind. For instance, the MetJ regulon, which controls methionine biosynthesis and transport, has been studied almost exclusively in *E. coli *and *Salmonella*, but homologs of MetJ exist in a variety of other species. These include some that are pathogenic (e.g. *Yersinia*) and some that are important for environmental remediation (e.g. *Shewanella*) but many of which have not been extensively characterized in the literature.

**Results:**

We have determined the likely composition of the MetJ regulon in all species which have MetJ homologs using bioinformatics techniques. We show that the core genes known from *E. coli *are consistently regulated in other species, and we identify previously unknown members of the regulon. These include the cobalamin transporter, *btuB*; all the genes involved in the methionine salvage pathway; as well as several enzymes and transporters of unknown specificity.

**Conclusions:**

The MetJ regulon is present and functional in five orders of gammaproteobacteria: Enterobacteriales, Pasteurellales, Vibrionales, Aeromonadales and Alteromonadales. New regulatory activity for MetJ was identified in the genomic data and verified experimentally. This strategy should be applicable for the elucidation of regulatory pathways in other systems by using the extensive sequencing data currently being generated.

## Background

The advent of fast sequencing techniques over the past few decades has led to an exponential increase in the number of fully sequenced organisms, particularly bacteria with their smaller genomes. Although a great deal of data is being generated, much of it is in the form of annotations based on automated similarity searches. It is important to validate these annotations and extend the use of the databases by organizing the genes into coherent pathways.

**Figure 1:**
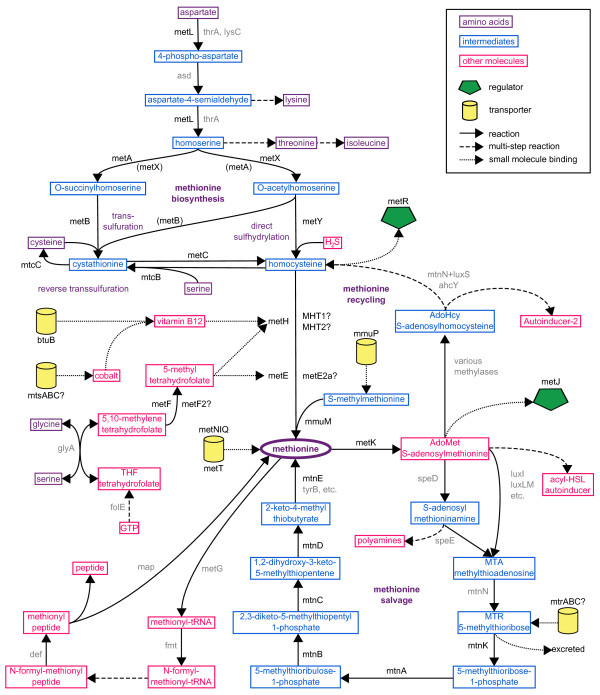
**Methionine as the center of various metabolic pathways**. Key is in upper-right. Genes regulated by MetJ are in black; other genes are in gray.

Here sequenced bacterial genomes were used to determine the full extent of the MetJ regulon in gammaproteobacteria. This regulon is controlled by the transcription factor MetJ which represses genes involved in methionine biosynthesis and transport [1-4]. Figure 1 shows various metabolic pathways involving methionine and Table 1 gives the functions of the genes. To date, the MetJ regulon has been studied almost exclusively in *E. coli *and the closely-related *Salmonella*, but it is likely that the complement of repressed genes extends beyond that known in these organisms.

**Table 1:** Functions of the genes involved in methionine utilization

gene	function^1^
**Regulators**	
*metJ*	AdoMet-dependent transcriptional repressor
*metR*	homocysteine-dependent transcriptional activator
**Biosynthetic enzymes**	
*metL*	aspartokinase II/homoserine dehydrogenase II
*metA*	homoserine O-succinyltransferase
*metX*	homoserine O-acetyltransferase
*metY*	~O-acetylhomoserine sulfhydrylase
*metB*	cystathionine gamma-synthase
*metC*	cystathionine beta-lyase
*metF*	5,10-methylenetetrahydrofolate reductase
*metH*	methionine synthase (B12-dependent)
*metE*	methionine synthase (B12-independent)
*metK*	S-adenosylmethionine (AdoMet) synthetase
**Methionine salvage pathway genes**	
*ybdH*	~alcohol dehydrogenase
*mtrA*	~MTR transporter (ATPase subunit)
*mtrC*	~MTR transporter (permease subunit)
*mtrB*	~MTR transporter (substrate-binding subunit)
*mtrY*	~function unknown
*mtnK*	5-methylthioribose (MTR) kinase
*mtnA*	S-methyl-5-thioribose-1-phosphate isomerase
*mtnD*	1,2-dihydroxy-3-keto-5-methylthiopentene dioxygenase
*mtnC*	2,3-diketo-5-methylthio-1-phosphopentane phosphatase
*mtnB*	methylthioribulose-1-phosphate dehydratase
*mtnE*	methionine aminotransferase
*mtnU*	~nitrilase
**Transporters**	
*metN*	methionine transporter (ATPase subunit)
*metI*	methionine transporter (permease subunit)
*metQ*	methionine transporter (substrate-binding subunit)
*metT*	~methionine transporter (Na+/H+ antiporter family)
*btuB*	vitamin B_12 _(cobalamin) outer membrane transporter
*mtsA*	~cobalt transporter (substrate-binding subunit)
*mtsB*	~cobalt transporter (ATPase subunit)
*mtsC*	~cobalt transporter (permease subunit)
*nhaP*	~Na+/H+ antiporter
**Other regulated genes**	
*mmuP*	S-methylmethionine permease
*mmuM*	S-methylmethionine : homocysteine methyltransferase
*MHT1*	~homocysteine S-methyltransferase
*dppA*	~transporter (substrate-binding subunit)
*dppB*	~transporter (permease subunit)
*dppC*	~transporter (permease subunit)
*dppD*	~transporter (ATPase subunit)
*MHT2*	~homocysteine S-methyltransferase
*arcD*	~amino acid permease
*metE2a*	~methionine synthetase (B12-independent)
*mtcB*	cystathionine beta-synthase
*mtcC*	cystathionine gamma-lyase
*megL*	~methionine gamma-lyase
*pcbC*	~oxygenase
**Various unregulated genes**	
*ahcY*	adenosylhomocysteinease
*asd*	aspartate semialdehyde dehydrogenase
*def*	peptide deformylase
*fmt*	10-formyltetrahydrofolate : L-methionyl-tRNAfMet N-formyltransferase
*folE*	GTP cyclohydrolase I
*glyA*	serine hydroxymethyltransferase
*luxS*	S-ribosylhomocysteine lyase
*map*	methionine aminopeptidase
*metG*	methionyl-tRNA synthetase
*mtnN*	methylthioadenosine (MTA) nucleosidase/S-adenosylhomocysteine (AdoHcy) nucleosidase

^1 ^Functions predicted from sequence similarity are marked with a tilde (~).

Previously, genes in the regulon were recognized on an individual basis by identifying DNA binding sites for MetJ, called metboxes, or by showing regulation via MetJ, methionine or its derivative S-adenosylmethionine (AdoMet) which is the co-factor for MetJ. Efforts to do large-scale, undirected searches for MetJ-regulated genes have been limited to *E. coli *[5,6]. By expanding our search to all sequenced bacteria, the dataset we had available for this study included 206 sequenced organisms representing 41 genera in 5 orders.

In this study, we have identified the conserved members that form the core of the regulon in all the organisms. While each species has a unique spectrum of regulated genes, the over-all regulon includes all the genes involved in methionine biosynthesis, salvage and transport. We have identified some previously unknown members of the regulon and, in addition, have experimentally confirmed that some of these are regulated by MetJ *in vivo*.

## Results

### MetJ homologs and metboxes

**Figure 2:**
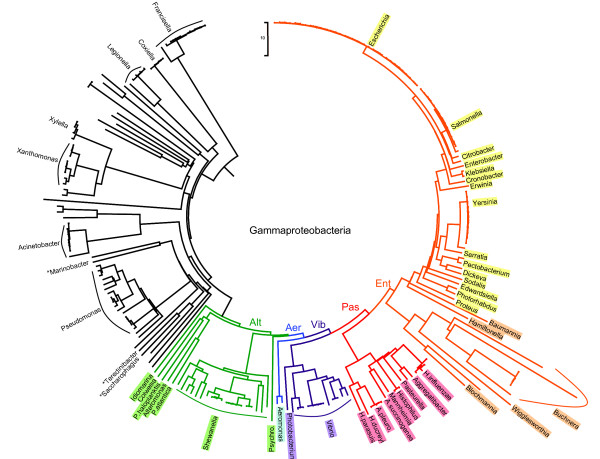
**Phylogenetic tree of the gammaproteobacteria**. Species with MetJ homologs are color-coded by order: Alt, Alteromonadales (green); Aer, Aeromonadales (blue); Vib, Vibrionales (purple); Pas, Pasteurellales (red); Ent, Enterobacteriales (orange for symbiotes, yellow for the rest). Selected other species are show in black. Three species marked with an asterisk were assigned to Alt but do not cluster with other Alt species.

Based on BLAST searches, MetJ homologs exist only within the gammaproteobacteria, and only within five orders: Enterobacteriales (Ent), Pasteurellales (Pas), Vibrionales (Vib), Aeromonadales (Aer) and Alteromonadales (Alt). These orders are shown in the phylogenetic tree in Figure 2 which was generated by the Orthologous Matrix Project based on whole genome comparisons of sequenced bacteria [7]. The most recent revision (December, 2010) includes most species that were part of this study. The gammaproteobacteria represent an important group of bacteria that are intimately involved in human life. They not only include the causative agents of diseases like cholera (*Vibrio cholerae*), chancroid (*Haemophilus ducreyi*), typhoid (*Salmonella enterica*) and the plague (*Yersinia pestis*), but they also include species that provide benefits such as aiding in the digestion of humans (*Escherichia coli*) and cows (*Mannheimia succiniciproducens*), acting as an agricultural insecticide (*Photorhabdus luminescens*), and being used in environmental bioremediation (*Shewanella oneidensis*). The five orders of interest all cluster in one part of the tree so it appears that MetJ entered the bacterial lineages at their common origin. All the sequenced organisms within these five orders have MetJ homologs with the exception of symbiotic Ent species (colored in orange). Almost all these species have lost MetJ as part of the massive reductions their genomes underwent during their shift to a symbiotic lifestyle. We note that three species (*Marinobacter aquaeolei*, *Saccharophagus degradans *and *Teredinibacter turnerae*) were annotated as Alt but they lack a MetJ homolog and metboxes, and they do not cluster with other Alt species, so they may have been misclassified. They were thus excluded from the analysis.

**Figure 3:**
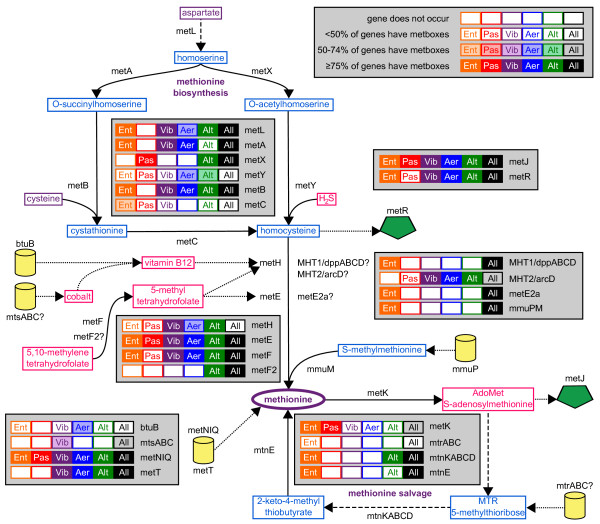
**Summary of genes regulated by MetJ in the various metabolic pathways**. The extent of regulation for each gene in the MetJ regulon is mapped onto a compressed pathway of methionine metabolism. Genes are annotated for their presence in an order, as well as the percent of those genes that have metboxes. Symbiotic bacteria have been excluded from "Ent" (Enterobacteriales), but are included in "All". Key is in upper-right; other features are the same as in Figure 1.

In order to look for metboxes in all these species, the known metboxes from *E. coli *were used as a training set to identify potential metboxes. High-confidence sequences that occurred with genes which were part of the known MetJ regulon in *E. coli *were used to create the final bit matrix for the final search, as described in the Methods. Methionine-related genes which had potential metboxes in a large number of species, particularly across multiple orders, make up the final list of candidate genes. Table 2 summarizes the membership in the MetJ regulon, indicating how many species within an order have the gene, and how many of those genes have metboxes. Figure 3 maps the degree of regulation for each gene onto the metabolic pathways involving methionine. A full list of candidate metboxes that were identified is available as Additional file 1 (Table S1), while a full list of candidate genes is available as Additional file 2 (Table S2). The spectrum of regulation shown in Table 2 and Figure 3 varies a great deal for each species, but, as expected, the known biosynthetic and transporter genes are well-represented.

**Table 2:** Composition of the MetJ regulon for the five orders

	Ent	Sym	Pas	Vib	Aer	Alt	All
species	122	16	20	16	3	29	206

*metJ*	*122 (122)	2 (0)	20 (15)	16 (16)	3 (3)	29 (28)	**192 (184)**
*metR*	*122 (122)	2 (1)	19 (3)	16 (16)	3 (3)	26 (22)	**188 (167)**

*metL*	122 (117)	--	--	16 (16)	3 (2)	26 (24)	**167 (159)**
*metA*	*122 (120)	3 (0)	--	16 (15)	3 (3)	27 (9)	**171 (147)**
*metX/1/2*	--	--	*19 (19)	--	--	--	**19 (19)**
*metY*	12 (0)	--	1 (0)	4 (0)	3 (2)	*25 (18)	**45 (20)**
*metB*	*122 (122)	3 (0)	--	16 (16)	3 (3)	25 (25)	**169 (166)**
*metC*	*119 (81)	3 (0)	19 (0)	16 (0)	--	24 (8)	**181 (89)**
*metF*	*122 (122)	13 (0)	18 (2)	16 (16)	2 (2)	23 (23)	**194 (165)**
*metF2*	--	--	--	--	--	5 (4)	**5 (4)**
*metH*	*112 (1)	--	9 (0)	*16 (16)	3 (2)	*28 (26)	**168 (45)**
*metE*	*122 (122)	13 (1)	17 (14)	16 (16)	3 (3)	19 (18)	**190 (174)**
*metK*	*122 (119)	14 (0)	20 (15)	16 (3)	3 (0)	29 (7)	**204 (144)**

*ybdH/2*	*101 (79)	--	--	--	--	--	**101 (79)**
*mtrA*	*41 (19)	--	--	--	--	--	**41 (19)**
*mtrC*	41 (19)	--	--	--	--	--	**41 (19)**
*mtrB/2*	*40 (26)	--	--	--	--	--	**40 (26)**
*mtrY/2*	31 (9)	--	--	--	--	--	**31 (9)**
*mtnK*	*38 (35)	--	--	1 (0)	--	1 (1)	**40 (36)**
*mtnA*	*38 (35)	--	--	--	--	1 (1)	**39 (36)**
*mtnD*	35 (29)	--	--	--	--	1 (1)	**36 (30)**
*mtnC*	35 (35)	--	--	--	--	22 (1)	**57 (36)**
*mtnB*	*35 (35)	--	--	--	--	2 (2)	**37 (37)**
*mtnE*	*105 (99)	--	--	--	--	3 (1)	**108 (100)**

*metNIQ*	*122 (121)	1 (1)	20 (16)	16 (16)	3 (3)	2 (2)	**164 (159)**
*metT*	--	--	--	*11 (11)	2 (2)	*26 (26)	**39 (39)**
*btuB*	*116 (34)	--	3 (0)	16 (6)	3 (2)	27 (6)	**165 (48)**
*mtsABC*	--	--	--	10 (6)	--	--	**10 (6)**
*nhaP*	--	--	10 (0)	*16 (16)	2 (2)	1 (0)	**29 (18)**

*mmuPM*	*21 (21)	--	--	--	--	--	**21 (21)**
*MHT1/dppABCD*	9 (9)	--	--	--	--	--	**9 (9)**
*MHT2/arcD*	--	--	5 (0)	1 (1)	2 (2)	6 (6)	**14 (9)**
*metE2a*	*28 (28)	--	--	--	--	--	**28 (28)**
*mtcBC*	20 (4)	--	--	--	--	2 (0)	**22 (4)**
*pcbC/megL*	--	--	--	--	2 (0)	5 (5)	**7 (5)**

The number of species within an order that have each gene are indicated and the number of those genes that have metboxes is in parentheses. Symbiotic Enterobacteria have been counted separately. Genes from an order that were tested experimentally for repression are marked with an asterisk.

For the entire complement of the MetJ regulon, most organisms regulate 70-80% of the genes, particularly Ent, Vib and Alt. For the Aeromonads, *A*. *hydrophila *regulates 94% of its genes, while *Tolumonas *only does 57%. The Pasteurellales are also highly variable, with repression percents ranging from 27% for *Aggregatibacter *to 69% for *Pasteurella*. Among the Ent symbiotes, only two species, *Hamiltonella defensa *and *Baumannia cicadellinicola*, have MetJ homologs, and it is a pseudogene in the latter. (We note that the classification of genes as pseudogenes in this paper is based on the genome annotation and therefore could be affected by sequencing errors. However in the case of symbiotic species, pseudogenization and gene loss are common features of reductive genome evolution [8].) These species are also the only symbiotes with metboxes, although with a limited distribution; in *H. defensa *only *metNIQ *has them, while in *B. cicadellinicola *only *metE/R *have them. The metbox regions for these genes are highly similar to those of other Ent species (70% identical to the corresponding regions of *E. coli*) and appear to be conserved from the common ancestor.

In addition to the Ent symbiotes, *Sodalis glossinidius *(Ent) and *Haemophilus ducreyi *(Pas) show a great deal of attrition in the MetJ regulon. *S. glossinidius *lives as a symbiote of tsetse flies but unlike the other symbiotic species, it has not reduced its genome significantly. However, while the genome is about the same size as *E. coli*, *S. glossinidius *has only about half as many intact genes. For our genes of interest, *metE2a*, *metH*, *metL*, *mtnK*, *mtnA*, *mtnC*, *mtnE *and *btuB *are identifiable as (unannotated) pseudogenes. Other than *metH *and *metL*, it retains the full complement of biosynthetic enzymes. In the case of *H. ducreyi*, an obligate pathogen, almost all genes of the MetJ regulon have been lost and it retains only *metJ*, *metK *and *metNIQ*. Of these, only *metK *retains its metboxes, so we may be seeing the first stage of the loss of *metJ *from this organism.

### Regulation by MetJ

In order to confirm their regulation by MetJ, some of the candidate genes were chosen for further study. The ability of MetJ to regulate the genes was assessed *in vivo *using an ONPG assay where the native operators (400 base pairs upstream of the coding sequence) were used to direct expression of *lacZ*. We cannot assume all foreign promoters will be functional in *E. coli*, but we are interested only in the expression of the gene in the presence of MetJ relative to the expression without MetJ, i.e. the repression ratio (RR). (We also note that we had difficulty transforming constructs containing *H. influenzae *operators into *E. coli*, although constructs from the other species could be readily transformed.)

**Figure 4:**
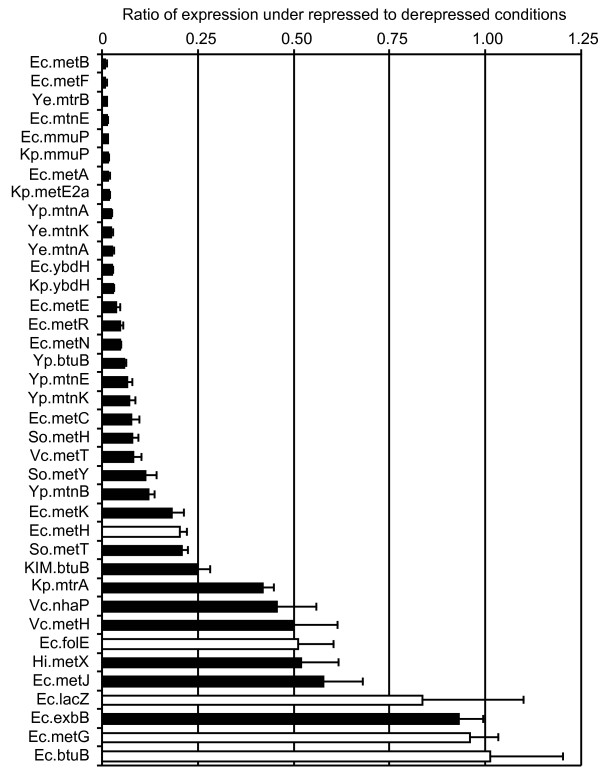
**ONPG assay for testing regulation *in vivo***. The results of the ONPG assay are summarized as the ratio of expression under repressed conditions (wt *E. coli*) to that under derepressed conditions (*ΔmetJ*). Black bars indicate the operator sequence was bound by MetJ in a gel-shift assay (Figure S1), while white bars were not bound. Species abbreviations are: Ec, *Escherichia coli *K12 W3110; Hi, *Haemophilus influenzae *Rd KW20; KIM, *Yersinia pestis KIM*; Kp, *Klebsiella pneumoniae*; So, *Shewanella oneidensis*; Vc, *Vibrio cholerae *N16961; Ye, *Yersinia enterocolitica*; Yp, *Yersinia pseudotuberculosis *YPIII.

We tested the known *E. coli *genes as well as some negative controls to determine the variability of repression. We then included a selection of genes from a variety of other organisms. Figure 4 shows the repression ratios for the various operators tested. The negative controls show ratios close to 1.00 while most of the repressed genes have ratios < 0.25. A gel shift was also performed to verify direct MetJ binding to the DNA sequences (Additional file 3 Figure S1), and those operators which were bound by MetJ are displayed as black bars in Figure 4. Ec.exbB is black because it shares an intergenic region with Ec.metC, but it is not regulated by MetJ *in vivo*. Ec.metH is white because it has no metboxes, but is strongly repressed *in vivo *by indirect regulation through MetR [9]. Ec.metJ itself shows fairly weak repression which is consistent with previous reports [10,11] and arises from the fact that one of its three promoters is constitutive and non-repressible. It is therefore likely that other genes with ratios around 0.50 are either weakly or indirectly repressed. Individual genes are discussed below.

### Transcription regulators

**Figure 5:**
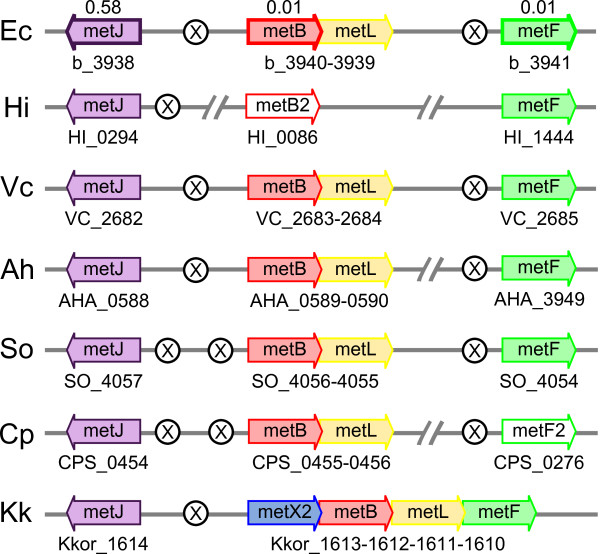
**Genomic organization of the genes in the *metJBLF *cluster**. Species are: Ec, *Escherichia coli *K12 MG1655 (Ent); Hi, *Haemophilus influenzae *Rd KW20 (Pas); Vc, *Vibrio cholerae *N16961 (Vib); Ah, *Aeromonas hydrophila *(Aer); So, *Shewanella oneidensis *(Alt); Cp, *Colwellia psychrerythraea *(Alt); and Kk, *Kangiella koreensis *(Alt). Genes are color-coded and spaces have been introduced between adjacent genes in order to align homologs vertically. In cases where a large number of other genes intervene between those of interest, a separation symbol is used://. Sites of metboxes are indicated by a circled X. Genes which were tested for repression in this paper are displayed with their repression ratio above the gene arrow.

MetJ is auto-regulated in 96% of the species where it occurs, although this repression is fairly weak. As described above, it was only repressed by half in our ONPG assay. In most species the gene occurs as part of a conserved cluster which includes *metB*, *metL *and *metF *(Figure 5), and it typically shares an intergenic region, and metboxes, with *metB*.

In addition to MetJ, some genes of the MetJ regulon are also regulated by MetR, a transcriptional activator of the LysR family. This protein primarily controls the expression of genes at the end of the pathway: *metF*, *metE *and *metH*, but also *metA*, *glyA*, *luxS *and *metR *itself. This gene is well represented in 91% of the species, and has metboxes in 89% of those. It almost always co-occurs with *metE *and they are divergently transcribed from a common intergenic region with common metboxes. In *Alteromonas macleodii*, an Alt species which lacks *metE*, *metR *is instead located 1 kilobase away from *metH*.

### Methionine biosynthesis genes: homocysteine formation

In the canonical biosynthetic pathway, the backbone of methionine comes from aspartate, which is also the precursor to lysine, threonine and isoleucine (Figure 1). Consequently, the three steps to convert aspartate to homoserine are catalyzed by the isoforms MetL (aspartokinase II, homoserine dehydrogenase II), ThrA (aspartokinase I, homoserine dehydrogenase I) and LysC (aspartokinase III), as well as the unique enzyme Asd (aspartate semialdehyde dehydrogenase). We note that all organisms without *metL *(mostly symbiotes and Pasteurellales) have *thrA *(distinguishable because it clusters with *thrB *and *thrC*) so it is possible that *thrA *provides the necessary function in those species. In almost every species of interest, the *metL *gene forms an operon with *metB*, and thus is regulated by the *metB *promoter (Figure 5). The only exceptions are *Idiomarina*, where *metB *has been lost, and *Kangiella*, where the operon has been expanded to include *metX2 *and *metF*.

The immediate precursor to methionine is homocysteine which can be made in multiple ways. Homoserine, created by MetL, is first activated by either succinylation or acetylation, and this represents the first committed step in methionine biosynthesis. These reactions are normally carried out by the unrelated proteins MetA (homoserine O-succinyltransferase) and MetX (homoserine O-acetyltransferase), however the two proteins do not have strict specificities [12]. Homocysteine can then be generated by either direct sulfhydrylation, a single step using inorganic sulfur, or by transsulfuration, a two step process using cystathionine as an intermediate with cysteine as the sulfur donor. The first reaction is usually catalyzed by MetY (O-acetylhomoserine (thiol)-lyase), while the second set of reactions is carried out by MetB (cystathionine gamma-synthase) and MetC (cystathionine beta-lyase). However, just as with MetA and MetX, there is a certain amount of promiscuity in the functions of MetY, MetB and MetC [13], particularly since they all belong to the same transsulfuration family of pyridoxal-dependent enzymes (Additional file 4 Figure S2).

For the first step of homoserine activation, almost all our species of interest use MetA (83%), and it has metboxes in most of them (86%). In addition, the *E. coli *protein is known to be feedback inhibited by methionine and AdoMet, and to be regulated at the level of transcription by MetR as well as MetJ. In most Ent and some Alt and Aer it occurs close in the genome to *metH*, the B_12_-dependent methionine synthase, even seeming to form an operon in *Pseudoalteromonas *and *Alteromonas *species.

The species without MetA either lack *de novo *biosynthesis (Ent symbiotes and *H. ducreyi*) or use MetX homologs (Pas and the Alt species *Idiomarina *and *Kangiella*). These genes all have metboxes. In addition, five Alt (three *Pseudoalteromonas*, *Alteromonas *and *Idiomarina*) and one Ent (*Pantoea *sp. At-9b) have a MetX homolog even though they also have MetA (or MetX1 in the case of *Idiomarina*). These genes do not have metboxes. The few *metX *genes that exist seem to have arisen from horizontal gene transfer; *metX *in Pas likely came from *Listeria *or a close relative [12].

In addition to replacing MetA with MetX, Pas species also replaced MetB with MetB2 from Gram-positives [14]. These two genes are not located close to each other on the chromosome so they may represent independent transfers. Possibly the native MetB was not capable of or efficient at recognizing the acetylated product of MetX; *E. coli *MetB can act on acetyl-homocysteine although not as efficiently as the succinylated substrate [13]. MetC remains to convert cystathionine to homocysteine. One of the *H. influenzae *strains (PittGG) lacks MetB2 and MetX is a pseudogene so it may have lost the ability to synthesize methionine. While all the Pas *metX *genes have metboxes and they were shown to be functional in *H. influenzae*, albeit weakly (RR = 0.52), only one of the *metB2 *genes has potential metboxes.

With the notable exception of Pas, succinylation by MetA and transsulfuration using MetB and MetC appears to be the ancestral pathway for homocysteine synthesis as these genes are well represented in all the orders. In almost all cases (98%) *metB *has metboxes, while *metC *has metboxes only in about half the Ent and a handful of Alt. Among the Ent symbiotes, only *Baumannia *and *Blochmannia *have the three genes, but none of them have metboxes. *metB *is a pseudogene in *Yersinia pestis *strains, which explains its known methionine auxotrophy [15]. Although it relies on transport to meet its methionine needs, *Y. pestis *has not yet lost any of the other genes associated with biosynthesis, or the metboxes that regulate them.

MetY homologs, which create homocysteine via direct sulfhydrylation, are eclectically represented in the five orders, but unlike MetA, MetB and MetC, the distribution of the MetY homologs does not match the phylogeny, so they are probably not inherited. There are metboxes in most of the Aer and Alt species and the gene from *Shewanella *is strongly repressed by MetJ (RR = 0.11). Almost all the species with MetY also have MetA, MetB and MetC so they may be able to use both pathways for homocysteine synthesis. The Alt *Idiomarina *is unique in that it lacks MetA, MetB and MetC and only has MetX1 and MetY, which form an operon and therefore presumably function together. *Kangiella *is odd in that it has *metB*, but not *metY *or *metC*. Probably MetB in this organism is capable of converting the MetX2 product directly to homocysteine through direct sulfhydrylation, a secondary function that *E. coli *MetB also has [13].

### Methionine biosynthesis genes: methionine formation

Homocysteine is converted to methionine using one of two unrelated enzymes: MetH, which requires vitamin B_12 _(cobalamin) as co-factor, and MetE. For organisms which have both proteins (~70% of our species), MetH is preferred when B_12 _is available since it is a much more efficient enzyme than MetE. MetH has metboxes in Vib, Aer and Alt species, but rarely in Ent or Pas. MetE, on the other hand, has metboxes in almost every species. This is probably because, in the absence of repression, the *metE *gene is very highly expressed to overcome the fact that it is such an inefficient enzyme [16]. In *E. coli*, this expression also requires MetR so it is not surprising that, with the exception of symbiotes, all species with *metE *also have *metR*, although the reverse is not true. As said before, *metE *typically shares a locus with *metR*, with the two genes being divergently transcribed from a common intergenic region in almost all species.

The terminal methyl group for methionine comes from the donor 5-methyltetrahydrofolate which is made by the enzyme MetF (5,10-methylenetetrahydrofolate reductase) and is usually in a glutamylated form. MetF has metboxes in almost all species (85%), the primary exception being Pas. In addition, there are four Alt species (*Psychromonas ingrahamii*, *Shewanella piezotolerans*, *Colwellia psychrerythraea *and *Pseudoalteromonas haloplanktis*) that lack the ortholog of *E. coli metF*, but instead have a gene we will refer to in this paper as *metF2*. This is only weakly similar to *metF*, but contains the same conserved domain (cd00537; PF02219). Because it occurs in species without *metF*, and because 3 of the 4 genes have metboxes, this gene likely performs the same function.

All but seven organisms have one or both of *metE *and *metH*, along with *metF *and thus can presumably synthesize methionine from homocysteine. Four of these deficient species are the symbiotes *Hamiltonella*, *Wigglesworthia*, *Riesia *and the Pas *H. ducreyi *which also lack the pathway for homocysteine synthesis. The Pas *A. succinogenes *and Alt *Idiomarina *lack *metE*, *metH *and *metF *while retaining the genes necessary for homocysteine synthesis. In the case of *Idiomarina*, it was shown that it is capable of growing in the absence of methionine [17] so it must have another pathway for its creation. It does not have any close homologs of *metH *or *metE*, but *A. succinogenes *has homologs of both *metH *(*MHT2*) and *metE *(*metE2b *and *metE2c*) so possibly one or more of these can complement the loss. The Aer *A. salmonicida *lacks only *metF *while retaining the synthases, so only its methyl donor is unknown.

### S-adenosylmethionine synthesis

Methionine can be further converted into S-adenosylmethionine (AdoMet) by the essential enzyme MetK. AdoMet is primarily used as a methyl donor but is also required for synthesis of polyamines, autoinducers and other molecules [18,19]. Crucially for the MetJ regulon, AdoMet is the co-factor for MetJ, which thus detects methionine levels only indirectly. The *metK *gene has metboxes in most Ent and Pas, but only a handful of Vib and Alt and no Aer. In many Gram-positive bacteria, which all lack MetJ, methionine biosynthesis is regulated by an AdoMet riboswitch [20,21]. Thus it seems that AdoMet synthesis is usually coordinately regulated as a part of methionine metabolism.

### Methionine salvage

The methionine salvage (MS) pathway allows the recycling of methylthioadenosine (MTA), produced during polyamine synthesis and other reactions, back to methionine (reviewed in [22]). MTA, which is a potent inhibitor of its parent reaction, is cleaved to methylthioribose (MTR) by the ubiquitous nucleosidase MtnN (5'-methylthioadenosine/S-adenosylhomocysteine nucleosidase). In *E. coli*, which lacks the MS pathway, MTR is excreted [23] and this may be true for other species which lack the pathway.

The enzymes which convert MTR to methionine in Enterobacteria are (in order of reaction): MtnK (5-methylthioribose kinase), MtnA (S-methyl-5-thioribose-1-phosphate isomerase), MtnB (methylthioribulose-1-phosphate dehydratase), MtnC (2,3-diketo-5-methylthio-1-phosphopentane phosphatase), MtnD (1,2-dihydroxy-3-keto-5-methylthiopentene dioxygenase) and MtnE (methionine aminotransferase). The pathway is illustrated in Figure 1. The final step can actually be catalyzed by multiple aminotransferases, such as *tyrB *in *Klebsiella *[24], while *mtnV*, the aminotransferase in *Bacillus subtilis*, was found to be dispensable [25]. Nevertheless, the *mtnE *gene is special because it only occurs in species that have (or whose ancestors had) the MS pathway, it clusters with other MS genes, and it is the only aminotransferase with metboxes. There are also some alternate proteins used by other species. For instance the single enzyme MtnP (methylthioadenosine phosphorylase) can be used in place of MtnN and MtnK, while MtnW (2,3-diketo-5-methylthiopentyl-1-phosphate enolase) and MtnX (2-hydroxy-3-keto-5-methylthiopentenyl-1-phosphate phosphatase) can be used in place of the bifunctional enzyme MtnC. None of these alternate enzymes have close homologs in our species of interest however.

In addition, these genes co-occur with a probable MTR transporter initially identified in *Bacillus cereus *[26]. It has not been functionally characterized yet, but is similar to other sugar transporters and has an S-box (AdoMet riboswitch) in *B. cereus *as well as metboxes in gammaproteobacteria. We will refer to the three components of this ABC transporter as *mtrACB*. The *mtrB *gene is usually closely followed by a gene of unknown function (COG5276) which we refer to as *mtrY *since its role is a mystery. Another gene, called *mtnU*, is closely associated with the MS genes. It is a nitrilase (from conserved domain cl11424) but its specificity is unknown. It was proposed to have a regulatory function in *B. subtilis *because it was produced at much lower levels than the other proteins [25]. Finally, another protein of unknown function, the putative alcohol dehydrogenase *ybdH*, also co-occurs with the MS genes and shares regulation by common intergenic metboxes.

**Figure 6:**
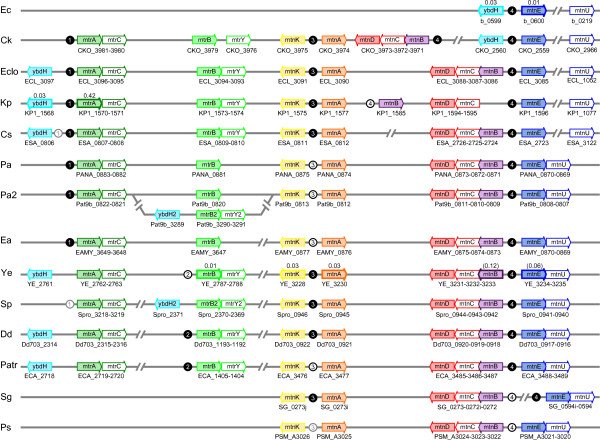
**Genomic organization of the genes of the methionine salvage pathway**. The different patterns are represented by one species for each genus with a unique pattern: Ec, *Escherichia coli *K12 MG1655; Ck, *Citrobacter koseri*; Eclo *Enterobacter cloacae *ATCC 13047; Kp, *Klebsiella pneumoniae *NTUH-K2044; Cs, *Cronobacter sakazakii*; Pa, *Pantoea ananatis*; Pa2, *Pantoea *sp. At-9b; Ea, *Erwinia amylovora*; Ye, *Yersinia enterocolitica*; Sp, *Serratia proteamaculans*; Dd, *Dickeya dadantii *Ech703; Patr, *Pectobacterium atrosepticum*; Sg, *Sodalis glossinidius*; Ps, *Pseudoalteromonas *sp. SM9913. Genes are color-coded and spaces have been introduced between adjacent genes in order to align homologs vertically. Pseudogenes in *S. glossinidius *have letters after the locus number. In cases where a large number of other genes intervene between those of interest, a separation symbol is used://. Groups of metboxes are indicated by circled numbers based on which genes they are adjacent to. These are filled in black if there is strong conservation (≥70% identity to a reference species), outlined in black for weaker conservation (≥60% identity), and outlined in gray for more divergent sequences. The reference species were *C. koseri *for groups 1, 3 and 4; and *P. atrosepticum *for group 2. Genes which were tested for repression in this paper are displayed with their repression ratio above the gene arrow. In the case of *Yersinia*, ratios in parentheses refer to *Y. pseudotuberculosis*.

Figure 6 shows the distribution and genomic context of the MS genes in our species of interest. Genes are color-coded and arranged to highlight the general conservation of the clusters. For all the MS genes tested, repression by MetJ was generally very tight (RR ≤ 0.12 for *ybdH*, *mtnA*, *mtnB*, *mtnK*, *mtnE *and *mtrB *in *E. coli*, *Yersinia *or *Klebsiella*, but 0.42 for *mtrA *in *Klebsiella*). The presence of metboxes between *ybdH *and *mtnE *(formerly *ybdL*) in *E. coli *was also noted by the Church group [27]; they generated knock-outs of the metboxes which caused an increase in the expression of both genes as determined by quantitative real-time PCR.

The full complement of genes exists only in the Enterobacteria, although individual species have suffered various rearrangements and losses. In *Klebsiella*, the *mtnB *gene was inverted and moved to the middle of a cluster of ~15 genes that were inserted between *mtnA *and *mtnD*. In the branch leading to *E. coli*, *Salmonella *and *Citrobacter *the entire cluster has been deleted, causing the flanking genes, *ybdH *and *mtnE*, to come together and share common regulation by the group 4 metboxes. In *C. rodentium*, even *ybdH *and *mtnE *were lost, but the upstream and downstream genes are the same as in the other species in that branch indicating that this was a localized deletion. In *C. koseri*, the entire MS cluster has then apparently been re-acquired, causing a duplication of the group 4 metboxes. For *Yersinia pestis*, all the strains except for Angola and Pestoides have lost the enzymes. They retain *ybdH *and the *mtrACBY *cluster, but *mtrA *is a pseudogene in all of them.

Oddly enough, one of the three *Pantoea *species (strain At-9b) has two *mtrB *genes. Pat9b_0820 occurs next to *mtrAC*, just like in other *Pantoea *and closely related *Erwinia *strains, while Pat9b_3290 occurs separate from the other MS genes in a cluster with *ybdH *and *mtrY*, which otherwise do not occur in *Pantoea *and *Erwinia*. Of all the other species with the MS pathway, only *Serratia *shares this pattern for the three genes. This suggests that in *Pantoea *sp. At9b, and maybe *Serratia *as well, the genes were re-acquired as a group. We note that none of the flanking genes are the same for these two species so the synteny only includes this 3-gene cluster. Since these genes also have different trees than the other MS genes, we distinguish them as *ybdH2*-*mtrB2*-*mtrY2*.

In addition, 3 of the 51 *Escherichia/Shigella *strains (UMN026, S88 and SE15) have acquired genes for *mtnK *and *mtnA*, apparently from alphaproteobacteria which have the most similar homologs. These genes form a cluster with a gene that is weakly similar to *mtnB*, but is most similar to the native *fucA *(D-ribulose-phosphate aldolase). We will refer to it as *mtnB2*. The insertion of the cluster was presumably phage-mediated as the genes are adjacent to a KpLE2 prophage. The genes co-occur with a fourth gene that is only found in these three *E. coli *strains and is annotated as a sugar permease. It is possible this cluster of genes is involved in the transport and enzymatic alteration of a sugar with unknown specificity. None of these genes have metboxes.

Enterobacteria at the base of the tree, *Edwardsiella*, *Photorhabdus*, *Proteus *and *Xenorhabdus*, lack all the genes associated with the MS cluster. The pathway is also lacking in the other orders, although some genes do unexpectedly show up in a handful of other species, e.g. an *mtnK *homolog in one Vib species (*Aliivibrio*). Among the Alt we find *mtnE *in two *Shewanella *species; *mtnC *in *Ferrimonas *and all 20 *Shewanella *species; *mtnB *and *mtnU *in *Pseudoalteromonas haloplanktis*; and the entire *mtn *cluster in *Pseudoalteromonas *sp. SM9913. Although we initially believed this was a result of horizontal gene transfer, phylogenetic trees for the genes match those of the organisms. Furthermore the metboxes, which are only preserved in the two *Pseudoalteromonas*, appear quite divergent from the Ent examples in the same context. This suggests that these genes have been in their genomes for a long time.

### Transporters

There are two known systems for methionine transport in the gammaproteobacteria. The most widespread is the ABC transporter MetNIQ which has been characterized in *E. coli *[3,4]. Except for two Alt species (*Psychromonas *and *Ferrimonas*), this transporter only occurs in Aer, Vib, Pas and Ent. The other transporter was recently identified in Gram-positive bacteria, as well as homologs in *Vibrio *and *Shewanella*, called MetT [26]. This is a member of the NhaC Na^+^/H^+ ^antiporter family and only occurs in Alt, Aer and Vib species. Thus *metT *appears to be the ancestral transporter that was lost at the time Pas branched off (see Figure 2), while *metNIQ *may be a newcomer that was introduced after most of the Alt species had already speciated. About 68% of Aer and Vib species retain both transporter systems. In almost all cases the transporter genes have metboxes and all the genes that were tested showed repression by MetJ: Ec.metN (RR = 0.05), Vc.metT (0.08) and Sh.metT (0.21). Thus all species, except for some symbiotes and two Alteromonads (*Alteromonas *and *Kangiella*), have methionine-specific transporters. In the case of *Alteromonas*, the only transporter with metboxes is MADE_2464 which is a putative polysaccharide transporter (conserved domain cl10513) and thus seems unlikely. In the case of *Kangiella*, one uncharacterized gene with potential metboxes has some similarity to dipeptide transporters: Kkor_1992 (DUF1302 superfamily). Homologs of this gene occur in other Alt species, but not with metboxes.

Vitamin B_12_, or cobalamin, is the necessary co-factor for the methionine synthase MetH, as well as some other enzymes. It is transported across the outer membrane by BtuB. Expression of this gene is known to be regulated by a riboswitch [28,29]. Regulation by MetJ was not suspected because *E. coli *and *Salmonella *lack metboxes. However, *btuB *genes in a third of our species of interest do have potential metboxes and the *Yersinia *ones we tested are functional (RR = 0.06 for *Y. pseudotuberculosis*, 0.25 for *Y. pestis *but 1.01 for *E. coli *which lacks the metboxes). The metboxes are located at the start of transcription, which includes the sizeable riboswitch, thus they are on average ~300 bp away from the start of translation. There are no other genes for which the metboxes are typically so far away. If it is the case the metboxes must precede riboswitches, then this suggests that no other gene in our study is also regulated by a riboswitch.

Another candidate transporter is similar to *mtsABC*, a metal transporter from Streptococcus [30]. This was also identified as part of the methionine regulon in Gram-positives [26]. Among our species, it only exists in Vib but has metboxes 60% of the time. It is annotated as a potential cobalt transporter which may link it to cobalamin utilization, just like *btuB*.

Finally, there is an NhaP Na^+^/H^+ ^antiporter family member in Pas, Vib, Aer and one Alt species. This is a transporter of unknown specificity. It has metboxes in some Vib and Aer species, but only when it shares an operator with *metH*. Although this pattern suggested that these metboxes should be specific to *metH*, when tested with the ONPG assay the *nhaP *gene from *V. cholerae *had the same level of repression as *metH *(0.46 vs. 0.50).

### Other regulated genes

There are various other genes that do not fit into the main categories of synthesis, salvage and transport. The *mmuPM *operon is composed of two genes: *mmuP *codes for an S-methylmethionine permease while *mmuM *codes for a specialized homocysteine methyltransferase that uses S-methylmethionine as its substrate [31,32]. MmuP is similar to other amino acid permeases like AroP and LysP while MmuM belongs to the same family as MetH. These genes exist in a random handful of Ent species. Among the 51 sequenced *Escherichia/Shigella *strains, only six K12 strains have homologs, and in those species they occur as part of the CP4-6 prophage. In strain BW2952, a subsequent deletion of ~100 kb led to the loss of *mmuM *and the fusion of the N-terminus of *mmuP *with the C-terminus of *mhpD*. This has happened recently enough that the metboxes have not been lost yet. For the other species, however, the homologs are not associated with a phage insertion. Furthermore, except for *Pectobacterium *the metboxes appear to be the same for all the species (≥60% identity to *Klebsiella*), suggesting a common origin for them. The *mmuPM *metboxes are unusual in that they occur very close to the start of translation, only 2 bases away in *E. coli*. For the other metboxes in this study, the average gap is ~75 bases. It was previously shown by immunoblotting that MmuM expression was reduced by high methionine concentrations [32]. We tested the operators for Ec.mmuP and Kp.mmuP and both were highly repressed (RR = 0.02) in the presence of MetJ.

**Figure 7:**
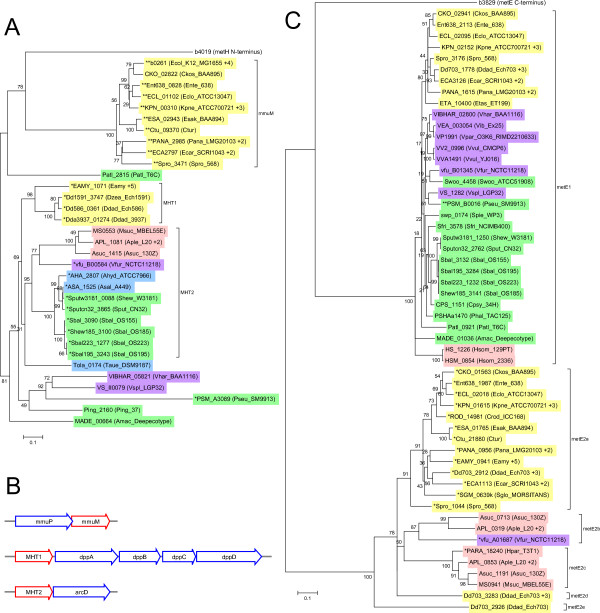
**Genes containing methionine synthase domains**. (A) Members of the MHT superfamily. Proteins were aligned with the first ~350 residues of *E. coli metH *which contains the MHT domain. (B) *mmuM*, *MHT1 *and *MHT2 *(in red) occur in clusters with transporters *mmuP*, *dppABCD *and *arcD *(in blue) respectively. (C) Members of the CIMS-C-terminal-like family. Proteins were aligned with the C-terminal half of *E. coli metE *which is the more conserved half. Genes have been collapsed by genus and are indicated by the species abbreviation of one representative member of the genus. The number in parentheses afterwards indicates the number of species which were excluded. Species color-coding is as described in Figure 2. Genes with metboxes are in bold and marked with an asterisk.

There are several homologs of the two methionine synthases, MetH and MetE. MetH (and MmuM) belong to the MHT (homocysteine/selenocysteine methylase) superfamily (conserved domain cl14105). All the closely related genes from our species of interest are shown in Figure 7A. These proteins correspond to the homocysteine-binding domain of MetH only and thus lack the tetrahydrofolate, B_12 _and AdoMet binding domains [33]. Like *mmuPM*, these genes co-occur with transporters and therefore may represent a specific transporter paired with a cognate enzyme (Figure 7B). There are two sub-groups that have metboxes: MHT1 is defined by the fact that these genes co-occur next to a putative peptide ABC transporter (similar to *E. coli dppABCD*) while MHT2 is defined by the genes' co-occurrence next to a member of the APC family of amino-acid transporters (similar to *arcD *from *Pseudomonas*). Among all the species of interest, the transporter genes only occur in these clusters with MHT homologs, making it more likely that they function together. We were not able to include these genes in the current study, but it would be desirable to test them later for activity and regulation.

MetE belongs to the CIMS-C-terminal-like family (conserved domain cd03311) and Figure 7C shows the other homologous genes. These proteins are half the size of MetE, which probably arose from an ancient duplication event [34]. Genes in the MetE1 group have no metboxes and occur downstream of a protein of unknown function (pfam08908) which only occurs with MetE1, suggesting these two proteins share a related function. None of the genes in the MetE2 cluster occur in a conserved environment, or adjacent to any putative transporter. Most of the genes in the MetE2a subgroup have metboxes. We tested *Klebsiella pneumoniae *and found that it was strongly repressed (RR = 0.02) by MetJ. Since all the organisms with MetE2a also have MetE, it is not clear what role this enzyme plays in metabolism.

In addition to the main MetNIQ cluster, there are various other components of this transporter system. There are six other 3-gene clusters with varying gene orders: *metNIQ*, *metIQN *and *metQNI*. These are referred to as metD2-7 in Table S2. There are two 2-gene clusters (*metNI*) and four isolated *metQ *homologs. (The number of homologs of *metN *was on the order of several thousand, so only those that cluster with *metI *or *metQ *are included in Table S2.) None of these genes are very common and very few of them have metboxes. MetQ is the periplasmic substrate-binding component and since MetNIQ can transport both D- and L-methionine, it was proposed that *metQ *and its only homolog in *E. coli*, called *nlpA*, could be specific for the different enantiomers [35]; unfortunately this was shown to be unlikely. However, some of the MetQ homologs in other species may show specificity.

Except for NlpA, none of the other MetNIQ homologs occur in *E. coli*, even though it is known to have a second methionine-specific transporter [36]. The high-affinity transporter, which can transport both D- and L-methionine, was called *metD *and was subsequently identified as *metNIQ*. The low-affinity transporter, which is specific to L-methionine, was called *metP *and is still unknown, although it was shown to not be *mmuP*, the S-methylmethionine permease [3,35]. Among the *E. coli *genes with metboxes, the only uncharacterized transporter is *yjhF *which is similar to gluconate transporters, but was shown not to be able to transport gluconate [37].

All the genes described above are likely members of the MetJ regulon. In addition, there are three other potential members that have functions related to methionine metabolism, but which we consider to be unlikely. The operon *mtcBC *encodes the genes involved in reverse transsulfuration -- the conversion of methionine to cysteine. MtcB is a cystathionine beta-synthase and is related to the cysteine synthases CysK and CysM. MtcC is a member of the transsulfuration family of enzymes, related to MetB, MetC and MetY. In our species of interest, these two genes only occur in 20 Ent and 2 Alt where they always occur together. Another potential member of the regulon is MegL. This is another transsulfuration family protein and is similar to methionine-gamma-lyase which is involved in methionine degradation [38,39]. There are several MegL-like genes, but only one group has metboxes. This group always clusters with an oxygenase similar to isopenicillin N synthase (PcbC, COG349).

For all these genes the number of homologs with metboxes is small. In the case of MegL only a few closely related *Shewanella *species have them. In addition to the paucity of metboxes, if the functional prediction is correct then it seems unlikely that they would be repressed by MetJ. They act to reduce methionine concentrations, so during times of MetJ activation (high methionine), these proteins should also be activated to allow for recyling of the sulfur group. Nevertheless, in *Klebsiella mtcBC *seem to be upregulated by MetJ, although the mechanism is not clear [14]. We include them as potential members of the regulon until they can be tested for actual regulation.

## Discussion

### The various regulons

The genes in the MetJ regulon in *E. coli *make up the core of the regulon that is consistently repressed in other gammaproteobacteria species. This includes all the genes involved in methionine biosynthesis and transport, although not every gene is regulated in every species. In addition, comparative genomics methods have identified other genes, such as the methionine salvage pathway, which does not occur in *E. coli*, but which is consistently regulated in all the species where it is found. With only a few exceptions, the methionine utilization genes that form the MetJ regulon in gammaproteobacteria are the same as those in Gram-positive bacteria, despite major differences in regulatory mechanisms [26].

Novel members of the regulon that we were able to verify experimentally are *btuB *(vitamin B_12 _transporter), *metX *(homoserine O-acetyltransferase), *metY *(possible O-acetylhomoserine sulfhydrylase), *metH *(B12-dependent methionine synthase), *metE2a *(possible methionine synthase), *mtnKADCB *(methionine salvage pathway enzymes), and the transporters *mtrACB*, *metT *and *nhaP*. We also identified some potential members that we were not able to test at this time: *metF2 *(possible 5,10-methylenetetrahydrofolate reductase), *MHT1 *and *MHT2 *(possible methionine synthases), *mtcBC *(reverse transsulfuration enzymes), *megL *(possible methionine gamma-lyase) and *mtsABC *(metal transporter). These novel genes highlight one of the major advantages of genomics-based methods since most of these genes do not exist in *E. coli*, and the others, like *btuB *and *metH*, are not regulated by MetJ.

There are some genes that appear never to have metboxes in the species we studied. These include *asd*, *glyA *and *folE *which are peripheral genes in the methionine biosynthetic pathway; *mtnN*, *luxS *and *ahcY *which are involved in recycling S-adenosylhomocysteine back to homocysteine; *metG*, *fmt*, *def *and *map *which generate methionyl-tRNA, formylate it, deformylate it and remove initial methionine from translated proteins; and the genes involved in cysteine metabolism. We note that *folE *was previously identified as a possible target for MetJ based on microarray data [6]. We could not identify metboxes for any of the *folE *homologs in the five orders and a gel-shift assay performed with *E. coli folE *showed no binding under our conditions (Figure S1). However, in the ONPG assay *folE *had weak repression so it may be indirectly regulated.

### The various organisms

The spectrum of regulated genes is undoubtedly related to the particular environments these organisms live in, as sulfur, and thus methionine, concentrations can vary a great deal [19]. One observation we can make from this study is that organisms which have developed an endosymbiotic (e.g. *Buchnera aphidicola *and *Sodalis glossinidius*) or obligate pathogenic (e.g. *Haemophilus ducreyi *and *Yersinia pestis*) lifestyle tend to lose regulon genes, as well as MetJ itself and the metbox sites. There are a variety of patterns of loss that likely reflect the degree of symbiosis as well as the time-scale involved. For instance the Ent symbiotes which lack MetJ have also lost all their metboxes and most regulon genes, while *Yersinia pestis *has a largely intact regulon with loss of only a few genes and no change in metbox distribution.

For free-living organisms there is likely to be a synergistic effect from the environmental niche in which the bacteria find themselves. For instance many Enterobacteria have a methionine salvage pathway which can utilize MTR as a sulfur source. In addition to endogenous production of MTR, these species likely can transport it from organisms like *E. coli *which excrete it into the common milieu. Likewise, S-methylmethionine is commonly produced by plants, and organisms with the *mmuPM *genes, like the plant pathogen *Pectobacterium*, can utilize this as a substrate for methionine synthesis.

### The various pathways

There are a variety of biosynthetic pathways on display in Figure 1, but there is clearly an ancestral pattern for methionine biosynthesis via MetA, MetB, MetC and the synthases MetE and MetH. Some organisms have replaced these with alternate pathways, such as MetX and MetB2 in Pas, or have acquired additional genes that may function in parallel, such as the many transsulfuration enzymes with unknown specificities. In particular, the spectrum of present, absent, regulated, and unregulated genes in Table S2 provides a rich resource for identifying non-canonical pathways or filling gaps in known ones. Alternate routes for methionine production may include reliance on transport of precursors, such as MTR and S-methylmethionine as well as homocysteine, or recycling pathways, such as methionine salvage which regenerates methionine molecules that derive their backbone from ribose rather than aspartate. For each species, a delineation of the full pathway would be necessary to understand the place of methionine biosynthesis within the metabolism of the whole organism. Such system-wide information is useful in metabolic engineering for production of organic molecules [40], as well as in the development of drugs for prokaryotic-specific proteins such as those of the methionine salvage pathway [41] and the cobalamin-independent methionine synthase MetE [42].

## Conclusions

This study is designed to use data from bacterial genome sequencing projects to gain a more complete understanding of the regulon controlled by MetJ in all the organisms in which it is found. We have identified over 2000 binding sites for ~35 genes and operons from 206 gammaproteobacteria species. Among these are genes expected from the *E. coli *MetJ regulon, as well as some new ones. For some of these genes we were also able to verify their regulation by MetJ experimentally.

## Methods

### Bioinformatics analysis

The genomes (and plasmids) for all sequenced species within the five orders containing MetJ homologs (Enterobacteriales, Pasteurellales, Vibrionales, Aeromonadales and Alteromonadales) were accessed from the Genome Information Broker [43,44] website in January, 2011. This was a total of 206 species. Orthologous genes were identified using BLAST searches [45], as well as consideration of the genomic environment. In most cases this identification was straight-forward since we were only interested in a subset of the gammaproteobacteria. However in some cases, particularly *metR *(part of the LysR family) and *btuB *(part of the TonB family), it was not always possible to confidently identify orthologs. For determining entire gene families (e.g. *metE *and *metH *in Figure 7), an e-value of e^-25 ^was used as the threshold. Gene alignments were made using ClustalW within MEGA5 [46]. Trees were made in MEGA5 using Neighbor-Joining with 500 bootstrap replicates. Data for the phylogenetic tree of gammaproteobacteria in Figure 2 was downloaded from the Orthologous Matrix Project [7,47] and manipulated in MEGA5.

### Metbox identification

An initial information weight matrix [48] was made using known metboxes for genes of the *E. coli *MetJ regulon (*metA*, *metJ/BL*, *metC*, *metE/R*, *metF*, *metK*, *metNIQ*, and *mmuPM*). The matrix is based on the information content of each base in each position of the metbox using the equation:

(1) $${\text{R}}_{\text{iw}}\left(\text{base},\text{position}\right)=\mu +\text{lo}{\text{g}}_{\text{2}}\text{ frequency}\left(\text{base},\text{position}\right)$$

Since the binding site is palindromic the data was symmetrized for each half-metbox.

This matrix was used to do a preliminary scan for metboxes in the 400-bp upstream of genes homologous to the *E. coli *MetJ regulon since those were likely to be real hits. One representative species from each genus was queried to reduce bias. To determine potential metboxes, a bit score was calculated for each 8-bp sequence by summing the values for the actual base at each position using the values in the matrix. Although each MetJ homodimer binds to a single 8-bp sequence, functional repression requires two or more adjacent sites [49]. A single metbox was required to have a bit-score greater than or equal to 1/4 the value of the consensus sequence, while the total site was required to have a bit-score greater than or equal to the value of the consensus sequence. These thresholds are arbitrary but were empirically found to be good at identifying known metboxes while reducing the number of false positives. The searches were performed using custom perl scripts. The 832 half-metboxes identified this way were used to create the final matrix (Table 3). This was then used to look for metboxes in the operators of all annotated genes in all species. The searches were not restricted to intergenic regions because of the possibility of incorrect automated annotation of genes. In practice, most of the high-confidence sites were located in intergenic regions, typically within ~75 bp of the start of translation. For determining whether particular metbox sequences are conserved or not, the metboxes were aligned and the percent of identical bases was determined relative to a reference species. The DNA compared include the metboxes as well as 8 basepairs on both sides since the flanking DNA is expected to vary more than the MetJ binding sites.

**Table 3:** Information weight matrix used to search for metboxes

	Count^1^				R_iw _(bits per base)^2^			
	1 (8)	2 (7)	3 (6)	4 (5)	1 (8)	2 (7)	3 (6)	4 (5)
**A**	**634**	156	**721**	37	**1.61**	-0.42	**1.79**	-2.49
**C**	6	25	84	**573**	-5.12	-3.06	-1.31	**1.46**
**G**	135	**560**	27	4	-0.62	**1.43**	-2.95	-5.70
**T**	57	91	0 > 1^3^	218	-1.87	-1.19	-7.70	0.07

^1 ^This refers to the number of times each base occurred at each position in the (symmetrized) metbox. The frequency used in Equation 1 is the count divided by the total number of half-metboxes: 832. The consensus sequence is highlighted in bold.

^2 ^R_iw _is the information content for each base at each position calculated using Equation 1. The consensus sequence is highlighted in bold.

^3 ^The occurrence of T (A) at position 3 (6) was zero, so this was changed to 1 to allow the log to be taken.

### *lacZ *reporter and ONPG assay

A reporter plasmid was made from pBR322 [50] by: removing the ampicillin resistance gene; making a silent mutation in the tetracycline resistance gene to remove an NheI site; and adding NsiI and KpnI sites by primer-amplification of the plasmid. The full-length *E. coli lacZ *gene sequence was amplified from strain W3110 with flanking NheI and KpnI sites, then cloned into the modified pBR322. This construct is referred to as pBZT. Operator sequences were cloned between the NheI and NsiI sites.

The candidate operators were cloned from one of eight bacteria: *Escherichia coli *W3110 and *Haemophilus influenzae *Rd KW20 (both courtesy C. Raetz, Duke University), *Klebsiella pneumoniae *and *Yersinia pestis *KIM (both courtesy S. Abraham, Duke University), *Yersinia pseudotuberculosis *YPIII and *Yersinia enterocolitica *8081 (both courtesy V. Miller, University of North Carolina at Chapel Hill), *Vibrio cholerae *N16961 (courtesy M. Kuehn, Duke University) and *Shewanella oneidensis *MR-1 (courtesy J. Frederickson, Pacific Northwest National Laboratory).

When necessary, genomic DNA was prepared by centrifuging an overnight culture of bacteria (1.5 ml) and resuspending the cells in 500 μl buffer containing 100 mM Tris, pH 8 and 50 mM EDTA. SDS was added to 1% to lyse the cells. 250 μl of 7.5 M ammonium acetate was added, then 500 μl phenol-chloroform. After spinning the sample down, the top 500 μl was removed and 1 ml isopropanol was added to precipitate the DNA. The DNA was washed with 70% ethanol, air-dried, then resuspended in 50 μl TE (10 mM Tris, pH 8, 1 mM EDTA).

Operator sequences were amplified by PCR from the genomic DNA using PfuUltra II HotStart 2× (Stratagene) with primers that produced flanking NsiI and NheI sites. (For operators which contained these sites, PstI and SpeI, respectively, were used instead to produce compatible ends.) These operators were cut and gel-purified then inserted into pBZT. All insertions were verified by sequencing. In cases where the insertion differed from the published sequence, a second independent construct was prepared to verify the polymorphism. In no case were these changes in the proposed metboxes. We note that our *Klebsiella *strain does not match any of the published genomes, but is most similar to *K. pneumoniae *NTUH-K2044.

Reporter plasmids were transformed into *E. coli *strain MC4100 (Δ(*argF-lac*)169) [51,52], which is missing the entire *lac *operon, and TK4100 (MC4100 *metJ::cam^r^*), a *metJ *knock-out strain (courtesy R. Greene, Duke University). Expression from the operator was measured using a β-galactosidase assay [53]. In brief, overnight cultures of the transformed strains were diluted 1/100 into fresh LB and grown for 2 hours at 37°C to mid-log phase (OD typically between 0.3 and 0.6). Aliquots of the cells were diluted into assay buffer (50 mM phosphate, pH 7, 100 mM NaCl and 1 mM DTT) and permeabilized with 10% toluene for at least 15 minutes at room temperature. ONPG was added to a final concentration of 1 mg/ml and the samples were then incubated at 37°C until they turned yellow, at which point they were quenched with an equal volume of 100 mg/ml sodium carbonate. Samples were spun down 5 min at max speed in a microcentrifuge, and the absorbance at 420 nm was then measured. The spectrometer was blanked against a parallel reaction made with untransformed MC4100. Miller units were calculated using the following equation:

(2) $$\text{1 Miller unit}=\text{Abs42}0÷\left[\text{vol}\left(\mu \text{l}\right)*\text{time}\left(\text{min}\right)*\text{OD6}00\right]$$

For each construct in each strain, at least two colonies were chosen, and triplicate samples were assayed for each colony. Results are represented as the ratio of the activity during repressed conditions (MC4100) to that during derepressed conditions (Δ*metJ*), and shown with the standard deviation for the six measurements.

### Gel shift assay

Operators were tested directly for MetJ binding using a gel-shift assay. For each plasmid, primers in the vector were used with MangoMix (Bioline) to amplify a 636-bp fragment incorporating the 400-bp operator. 50 μl PCR reactions were cleaned up using SureClean Plus (Bioline) and the precipitated DNA was resuspended to the same starting volume with TES Buffer (20 mM Tris, pH 8, 1 mM EDTA, 150 mM NaCl). 1 μl of the PCR mix (approximately 10 ng DNA) was used in a 30 μl reaction with 0 or 50 nM MetJ. Samples were run on a 7.5% TGE gel (25 mM Tris, 192 mM glycine, and 2 mM EDTA) containing 250 μM AdoMet, then stained with Vistra Green (GE Healthcare) and imaged with a Typhoon 9410 phosphorimager using a 520 BP 40 fluorescence filter.

## Authors' contributions

AMA conceived of the study, did the bioinformatics and biochemical analysis and drafted the manuscript. LDS assisted in study coordination, periodically reviewed results and helped draft the manuscript. All authors read and approved the final manuscript.

## Supplementary Material

Additional file 1 **Table_S1_List_of_candidate_metboxes**. Spreadsheet of all likely metboxes found in this study. Click here for file

Additional file 2 **Table_S2_List_of_loci_for_MetJ_regulon_genes**. Spreadsheet of all genes that form the MetJ regulon in all species, whether or not they have metboxes. Click here for file

Additional file 3 **Figure_S1_Gel_shift**. Gel shift assay showing MetJ binding to operator DNA. Each lane is ~10 ng DNA with or without 50 nM MetJ in the presence of 250 μM AdoMet. Genes are indicated by locus number and those which share an operator are linked by a brace. The molecular weight marker is GeneRuler 1 kb Plus DNA Ladder (Fermentas). Click here for file

Additional file 4 **Figure_S2_Tree_of_transsulfuration_enzymes**. Tree of transsulfuration enzymes. Genes have been collapsed by genus and are indicated by the species abbreviation of one representative member of the genus. The number in parentheses afterwards indicates the number of species which were excluded. Species color-coding is as described in Figure 2. Genes with metboxes are in bold and marked with an asterisk. Click here for file
