# Supplementary material for: The MetJ regulon in gammaproteobacteria determined by comparative genomics methods
Source: BMC Genomics. 2011 Nov 14;12:558. doi: 10.1186/1471-2164-12-558 (PMC3228920; doi:10.1186/1471-2164-12-558)
Supplement: Additional file 3 — Figure_S1_Gel_shift. Gel shift assay showing MetJ binding to operator DNA. Each lane is ~10 ng DNA with or without 50 nM MetJ in the presence of 250 μM AdoMet. Genes are indicated by locus number and those which share an operator are linked by a brace. The molecular weight marker is GeneRuler 1 kb Plus DNA Ladder (Fermentas). [file 1471-2164-12-558-S3.PDF]

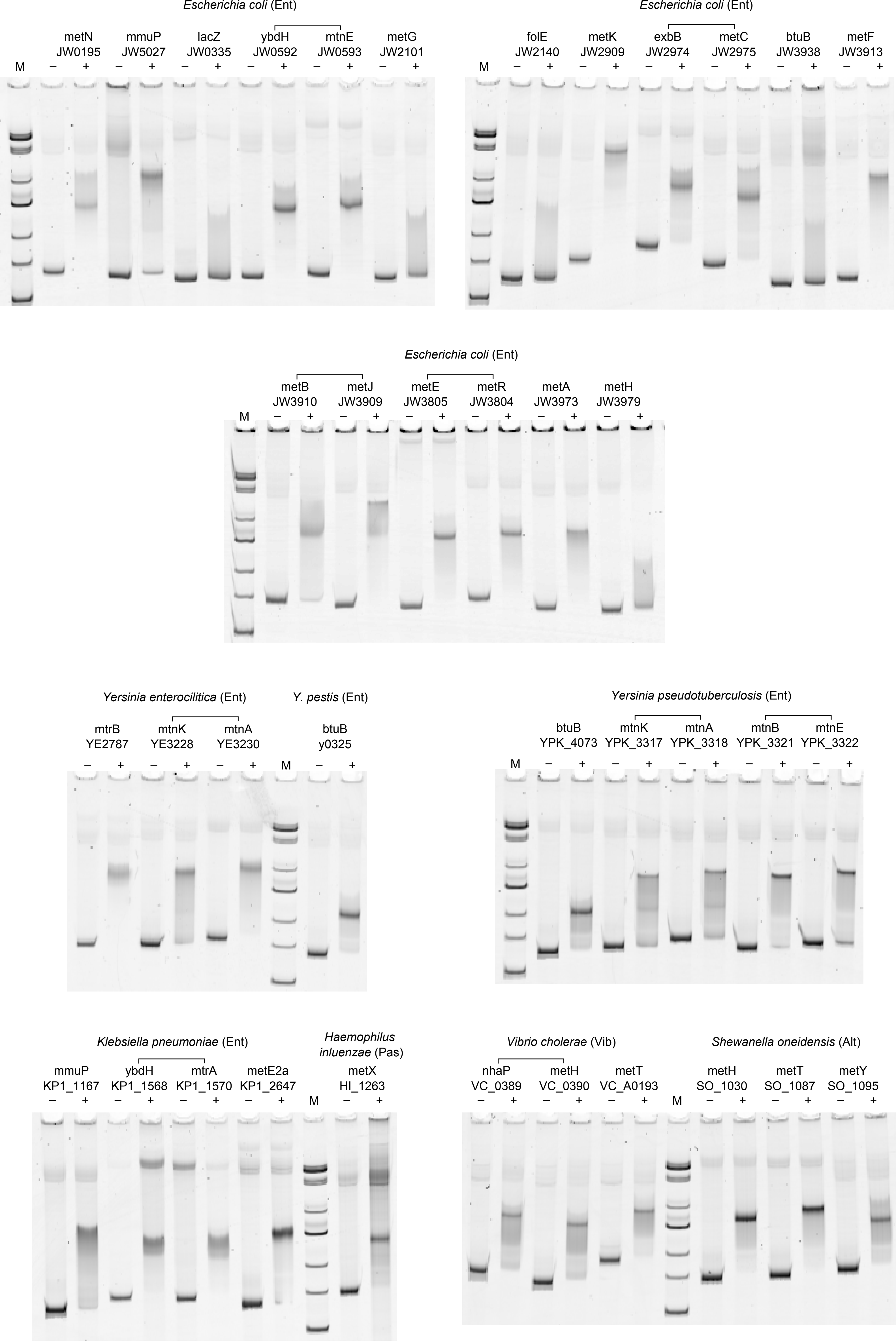
Escherichia coli (Ent)
metN JW0195
mmuP JW5027
lacZ JW0335
ybdH JW0592
mtnE JW0593
metG JW2101
M
– +
Escherichia coli (Ent)
folE JW2140
metK JW2909
exbB JW2974
metC JW2975
btuB JW3938
metF JW3913
Escherichia coli (Ent)
metB JW3910
metJ JW3909
metE JW3805
metR JW3804
metA JW3973
metH JW3979
Yersinia enterocilitica (Ent)
mtrB YE2787
mtnK YE3228
mtnA YE3230
Y. pestis (Ent)
btuB y0325
Yersinia pseudotuberculosis (Ent)
btuB YPK_4073
mtnK YPK_3317
mtnA YPK_3318
mtnB YPK_3321
mtnE YPK_3322
Klebsiella pneumoniae (Ent)
mmuP KP1_1167
ybdH KP1_1568
mtrA KP1_1570
metE2a KP1_2647
Haemophilus inluenzae (Pas)
metX HI_1263
Vibrio cholerae (Vib)
nhaP VC_0389
metH VC_0390
metT VC_A0193
Shewanella oneidensis (Alt)
metH SO_1030
metT SO_1087
metY SO_1095
