# Supplementary figures and images for: The MetJ regulon in gammaproteobacteria determined by comparative genomics methods
Source: BMC Genomics. 2011 Nov 14;12:558. doi: 10.1186/1471-2164-12-558 (PMC3228920; doi:10.1186/1471-2164-12-558)

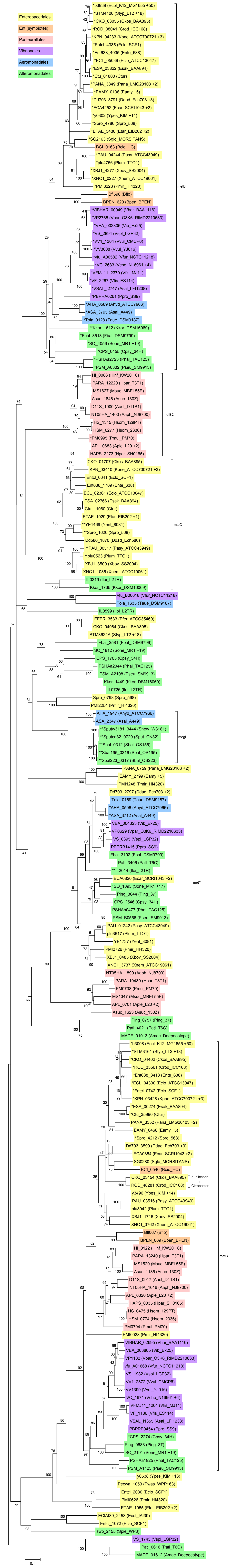

Supplement: Additional file 4 — Figure_S2_Tree_of_transsulfuration_enzymes. Tree of transsulfuration enzymes. Genes have been collapsed by genus and are indicated by the species abbreviation of one representative member of the genus. The number in parentheses afterwards indicates the number of species which were excluded. Species color-coding is as described in Figure 2. Genes with metboxes are in bold and marked with an asterisk. [file 1471-2164-12-558-S4.PDF]
